# Excellent survival in relapsed stage I testicular cancer

**DOI:** 10.1186/s12885-023-11388-y

**Published:** 2023-09-15

**Authors:** Philip Speicher, Christian D. Fankhauser, Anja Lorch, Davide Ardizzone, Simon Helnwein, Dennis Hoch, Thomas Hermanns, Jörg Beyer, Dilara Akhoundova

**Affiliations:** 1 Department of Medical Oncology and Hematology, Hospital of Thun, 3600 Bern, Switzerland; 2 Department of Medical Oncology, Inselspital, Bern University Hospital, University of Bern, 3010 Bern, Switzerland; 3 Department of Urology, Lucerne Cantonal Hospital, 6000 Luzern, Switzerland; 4 Department of Medical Oncology and Hematology, University Hospital Zurich, 8006 Zurich, Switzerland; 5 Faculty of Medicine, University of Zurich, 8006 Zurich, Switzerland; 6 Department of Urology, University Hospital Zurich, Zurich, Switzerland; 7 Department for Biomedical Research, University of Bern, 3008 Bern, Switzerland

**Keywords:** Testicular cancer, Germ-cell cancer, Clinical stage I, Active surveillance, Follow-up, Relapse, IGCCCG prognostic group

## Abstract

**Background:**

Two thirds of patients with germ-cell cancer (GCC) present as clinical stage I (CSI). Following orchiectomy, active surveillance (AS) has become their standard management. However, 15–50% of patients eventually relapse with metastatic disease after AS. Relapses need to be detected early in order to achieve cure and avoid overtreatment.

**Methods:**

We retrospectively analyzed consecutive GCC patients treated at two Swiss academic centers between 2010 and 2020. Patients with stage IS and extragonadal primaries were excluded. We compared disease characteristics and survival outcomes of patients relapsed from initial CSI to patients with de novo metastatic disease. Primary endpoint was the IGCCCG category at the time of relapse. Main secondary endpoints were progression-free survival (PFS) and overall survival (OS).

**Results:**

We identified 360 GCC patients with initial CSI and 245 de novo metastatic patients. After a median follow-up of 47 months, 81 of 360 (22.5%) CSI patients relapsed: 41 seminoma (Sem) and 40 non-seminoma (NSem) patients. All Sems relapsed in the IGCCCG good prognosis group. NSem relapsed with good 29/40 (72.5%) and intermediate 11/40 (27.5%) prognostic features; 95.1% of relapses occurred within five years post-orchiectomy. Only 3 relapsed NSem patients died from metastatic disease. Five-year OS for relapsed CSI patients was 100% for Sem and 87% (95% CI: 61–96%) for NSem patients; five-year PFS was 92% (95% CI: 77–97) and 78% (95% CI: 56–90) for Sem and NSem, respectively. When stratified by IGCCCG prognostic groups, good risk relapsed patients had a trend towards better OS and PFS as compared to de novo metastatic patients.

**Conclusions:**

GCC patients who relapse after initial CSI can be detected early by active surveillance and have an excellent survival.

**Supplementary Information:**

The online version contains supplementary material available at 10.1186/s12885-023-11388-y.

## Introduction

Germ-cell cancer (GCC) is the most common cancer in young men in North America and Europe and presents as clinical stage I disease (CSI) localized to one or both testicles in about 70% of patients. Active surveillance has become the standard management in CSI GCC. However, about 15–50% of patients eventually relapse with metastatic disease depending on size of the primary tumor, histology and other risk factors present in the primary tumor [1]. While the NCCN and EUA guidelines recommend AS as preferred option in patients with high-risk seminoma (Sem), the NCCN and EUA guidelines recommend AS as preferred option, the ESMO guidelines consider both, adjuvant chemotherapy with one cycle of carboplatin and AS as equally valid options [2–4]. For high-risk non-seminomas (NSem), the NCCN guidelines propose either AS, adjuvant chemotherapy with one cycle of bleomycin, etoposide and cisplatin (BEP) or retroperitoneal lymph node dissection (RPLND) as possible alternatives, while the ESMO and EUA guidelines recommend one cycle BEP [2–4].

Patients with relapses from CSI are managed identically to patients with de novo metastatic disease according to the prognostic classification of the International Germ Cell Cancer Consensus Group (IGCCCG) [5, 6]. Fundamental to the concept of active surveillance is that relapses are detected early in order to minimize treatment intensity at relapse and secure high survival probabilities [4, 7, 8]. However, data indicates that active surveillance recommendations are not uniformly followed [1]. We studied relapses from initial CSI GCC in consecutive patients from two Swiss university centers and compared their outcomes to patients with de novo metastatic disease.

## Methods

### Patients

We identified consecutive patients diagnosed with GCC at the University Hospitals Bern and Zurich (Switzerland) between 2010 and 2020. Subsequently, we reviewed all medical records of identified patients to capture all staging, treatment and follow-up clinical information using structured paper-based case report forms. Attempts were made to obtain missing clinical information by contacting referring or follow-up institutions. All captured data was subsequently entered by one of the authors (PS) into a central SPSS database (IBM SPSS Statistics, IBM Corp., Chicago, IL, USA, Version 28.0.1.1). Plausibility checks and extensive data cleaning was performed by two of the authors (AL and JB) prior to analysis to correct entry errors. The database was locked to entries on April 13th 2023.

Staging information included site and histology of the primary tumor, locations of metastases, serum tumor marker levels pre-chemotherapy, IGCCCG prognostic group, number of cycles and type of chemotherapy. Normal serum tumor marker levels were defined as alpha-fetoprotein (AFP) less than 10 μg/L and human chorionic gonadotropin (HCG) less than 5 U/L. For serum-LDH values, 250 U/l was selected as upper limit of normal (ULN). A multiplication factor of 1.5xULN was used as threshold to classify patients into the IGCCCG intermediate risk group [9].

### Endpoints and statistical analysis

Primary endpoint was the IGCCCG prognostic group of relapsing CSI patients. Secondary endpoints consisted of time to relapse, as well as progression-free survival (PFS) and overall survival (OS) probabilities. Primary and secondary endpoints of relapsing CSI patients were compared to patients with de novo metastatic GCC. PFS survival started at the first day of chemotherapy and ended at the day of documented progression or death. Progression was defined as a serological or radiological progression whichever occurred first. OS started at the first day of chemotherapy and ended the day of the last follow-up visit. A patient was declared lost to follow-up if we were unable to get information about his follow-up status despite contacting follow-up institutions, or if he did not return to the follow-up institution for further visits. Patients lost to follow-up were censored at the time of their last contact.

Descriptive statistical analyses were performed for categorical and continuous parameters of interest. PFS and OS probabilities were analyzed using the Kaplan–Meier method. Significance for survival analyses was tested using the log-rank test. A two-sided *p*-value of < 0.05 was considered significant. All tests were performed using the SPSS (IBM SPSS Statistics, IBM Corp., Chicago, IL, USA, Version 28.0.1.1) and STATA (StataCorp LLC, College Station, TX, USA, Version 10.1, 2008) software packages. The study was approved by the local ethical committee (BASEC ID 2023–00364).

## Results

### Patient characteristics

**Fig. 1:**
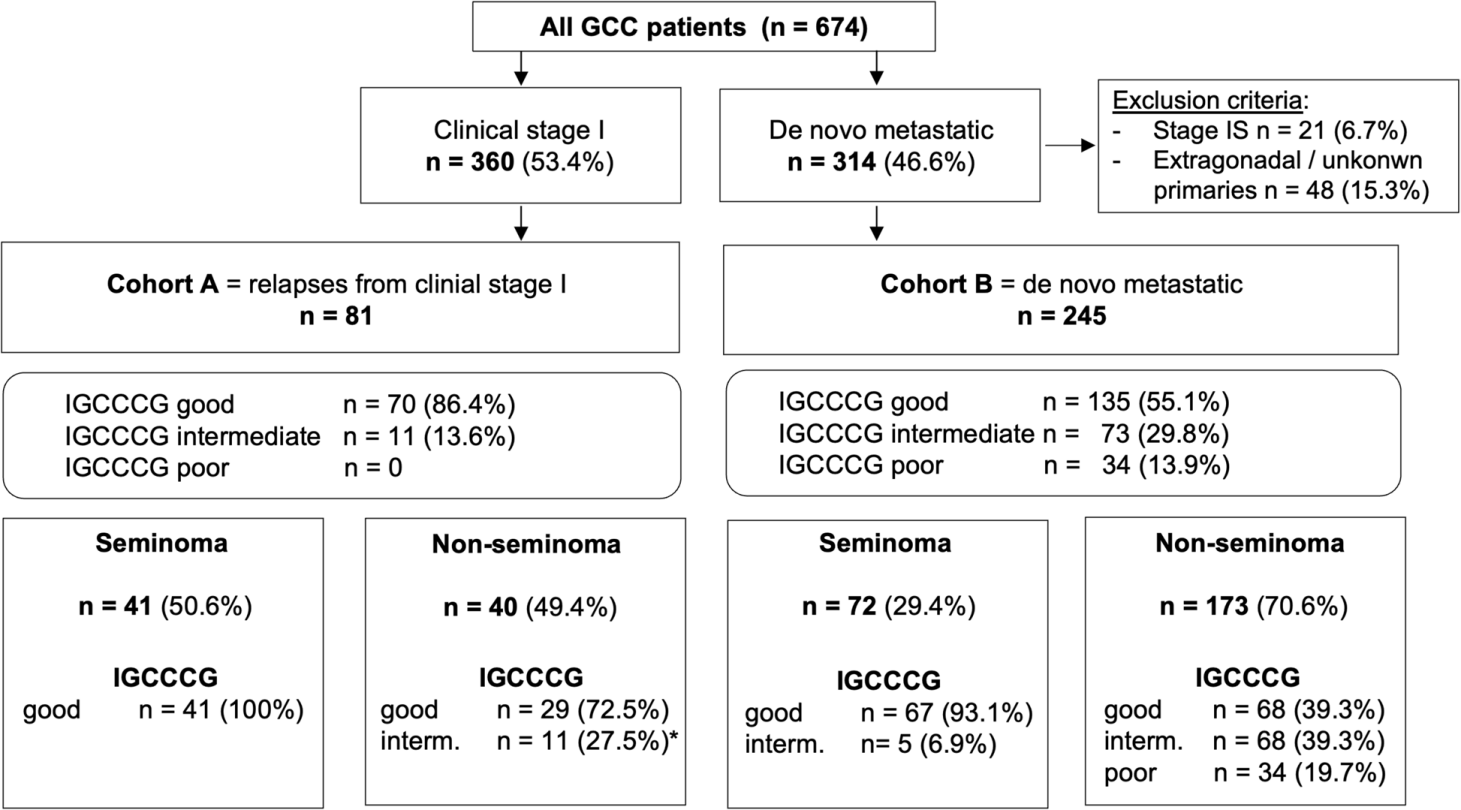
Schematic diagram illustrating patient distribution into cohorts A and B, as well as stratification by IGCCCG prognostic groups and histology. Abbreviations: GCC: germ cell cancer; IGCCCG: International Germ Cell Cancer Cooperative Group; *For serum-LDH values, 250 U/l was selected as upper limit of normal (ULN). 1.5xULN threshold was used to classify patients into the IGCCCG intermediate risk group

**Fig. 2:**
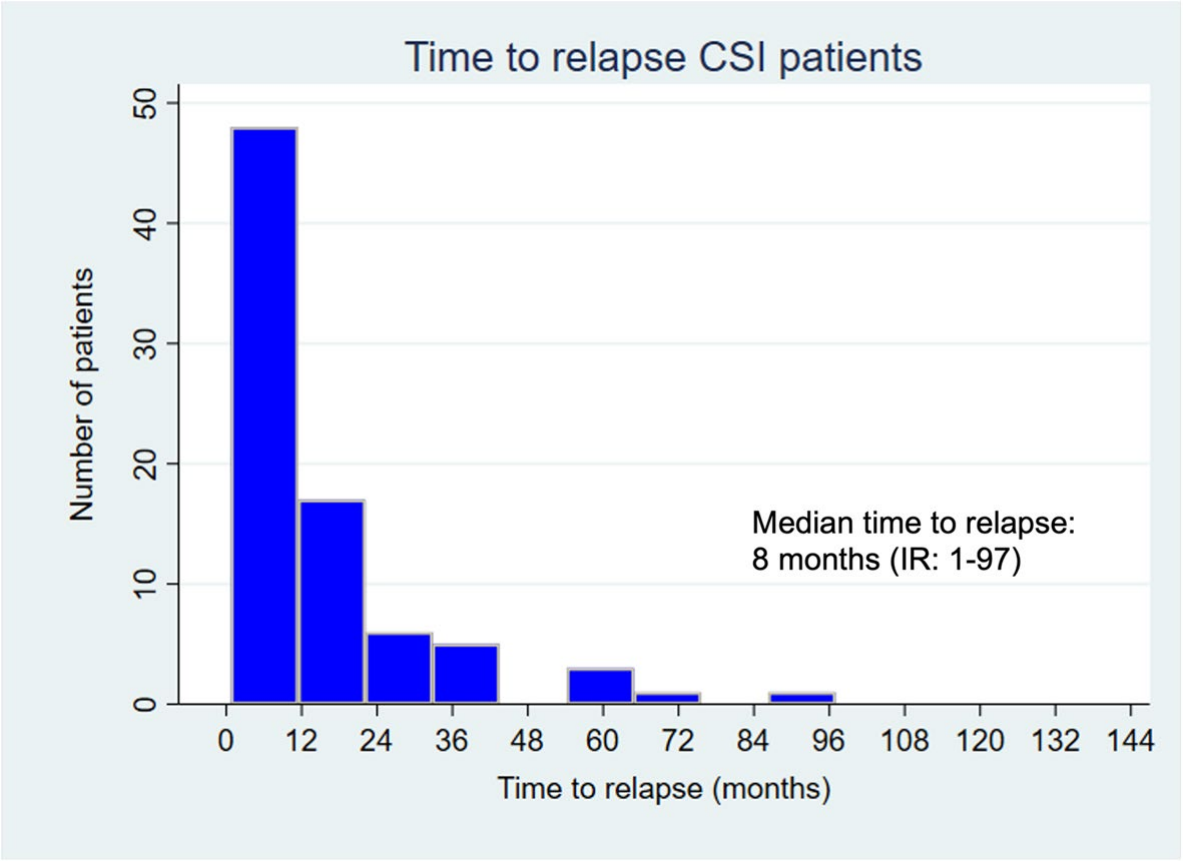
Histogram illustrating time to relapse (months) after initial CSI management. 95.1% of relapses occurred within five years post-orchiectomy

We identified 360 (53.4%) stage I and 314 (46.6%) de novo metastatic GCC patients referred to the University Hospitals Bern and Zurich between 2010 and 2020. 65.2% of CSI Sem patients and 73.0% of NSem patients were initially managed with active surveillance post-orchiectomy (Suppl. Figure 1). 81/360 (22.5%) of CSI patients relapsed after a median relapse-free survival of 8 months (interquartile range (IQR): 5 – 18.5). For the purpose of further analysis, we grouped the 81 relapsed patients as Cohort A. After excluding patients with stage IS (*n* = 21) and extragonadal or unknown primaries (*n* = 48), we grouped the remaining 245 de novo metastatic GCC patients as cohort B (Fig. 1, Supp. Figure 1). One third (16/48) of extragonadal GCC presented with mediastinal primary. 77 out of 81 (95.1%) relapses occurred within the first five years from initial CSI management (Fig. 2).

Patient baseline characteristics are summarized in Table 1. Median age at diagnosis was 33 vs 34 years in cohort A and B, respectively. Pure Sem histology was more frequent in Cohort A (50.6% vs 29.4%). Lymphovascular invasion and invasion of the rete testis were present in 26.7% and 46.3% of cases in the cohort A, and 55.8% and 49.5% of cases in the cohort B, respectively. After a median follow-up of 47 months (IQR: 23–71), 41/81 (50.6%) Sem and 40/81 (49.4%) NSem patients relapsed. Serum tumor marker values before treatment initiation for metastatic disease were numerically higher in Cohort B. All relapsed Sems were classified within the IGCCCG good prognosis group and 11 (27.5%) NSem relapses within the intermediate group based on an elevated LDH > 1.5 × upper limit of normal (ULN). Seven NSem patients in cohort A had missing LDH-values and were classified as good-risk based on favorable clinical parameters (Table 1, Fig. 1, Suppl. Figure 1). Among relapsed NSem patients 14 (35%) had lymphovascular invasion. Among relapsed Sem patients 21 (51.2%) had rete testis invasion and 15 (36.6%) a tumor size > 4 cm. Among the 107 CSI patients who received adjuvant chemotherapy (carboplatin or BEP), 14 (13.1%) patients relapsed (6 NSem (17.1%) and 8 Sem (11.1%). Median time to relapse among patients who received adjuvant chemotherapy was 19.5 months and no patients relapsed after the 5-year benchmark. Out of these 14 patients, 2 (14.3%) died due to disease progression, potentially suggesting that patients who still relapse despite adjuvant treatment have probably a more aggressive tumor biology. Among the 6 relapsed NSem, 1 patient had lymphovascular invasion, and, among the 8 relapsed Sem, 6 (75%) patients had rete testis invasion and 3 (38%) patients had a tumor size > 4 cm.

**Table 1:** Patient characteristics, treatment regimens and treatment responses

	**Cohort A** **(*n* = 81)**	**Cohort B** **(*n* = 245)**
**Age at diagnosis (year), median (range)**	33 (16 – 66)	34 (17 – 69)
**Histological subtype in primary tumor, number (%)**^**a**^		
Pure seminoma	41 (50.6%)	72 (29.4%)
Non-seminoma / mixed germ cell tumor	40 (49.4%)	161 (65.7%)
Pure Teratoma	0	10 (4.1%)
**Serum tumor markers prior to treatment, number (%)**^**b**^		
AFP median (range)	3.1 (n – 104)	4.5 (n – 63951)
< 1000 ng/ml	71 (100%)	210 (91.7%)
> 1000 and < 10000 ng/ml	0	14 (6.1%)
> 10000 ng/ml	0	5 (2.2%)
Missing values	10/81 (12.3%)	16/245 (6.5%)
HCG median (range)	0 (n – 549)	8 (n-1′995′937)
< 5000 IU/l	73 (100%)	200 (86.6%)
> 5000 and < 50′000 IU/l	0	14 (6.1%)
> 50′000 IU/l	0	17 (7.4%)
Missing values	8/81 (9.9%)	14/245 (5.7%)
LDH median (range)	360.5 (n – 1038)	416 (n – 5666)
< 1.5 × ULN	40 (60.6%)	73 (34.8%)
> 1.5 × and < 10 × ULN	26 (39.4%)	129 (61.4%)
> 10 × ULN	0	8 (3.9%)
Missing values	15/81 (18.5%)	35/245 (14.3%)
**IGCCCG risk classification, number (%)**^**c**^		
Good prognosis	70 (86.4%)	135 (55.1%)
Intermediate prognosis	11 (13.6%)^d^	73 (29.8%)
Poor prognosis	0	34 (13.9%)
**Treatment for metastatic disease**		
BEP	55 (67.9%)	174 (71.0%)
EP	7 (8.6%)	27 (11.0%)
VIP / TIP	3 (3.7%)	17 (6.9%)
Other^e^	9 (11.1%)	14 (5.7%)
Surgery	7 (8.6%)	0
Missing data	0	9 (3.7%)
Residual tumor resection post chemotherapy	12 (14.8%)	82 (34.5%)
Radiotherapy post chemotherapy	10 (13.2%)	13 (5.4%)
**Treatment response**		
Complete response (CR)	54 (68.4%)	77 (33.2%)
CR after residual tumor resection (CRar)	13 (16.4%)	59 (25.4%)
Partial response (PR)	11 (13.9%)	90 (38.8%)
Stable disease (SD) / Progressive disease (PD)	0	6 (2.6%)
Missing data	1 (1.2%)	13 (5.3%)

*Abbreviations*: *AFP* alpha-fetoprotein, *BEP* bleomycin, etoposide, cisplatin, *EP* etoposide, cisplatin, *TIP* paclitaxel, ifosfamide, cisplatin, *ULN* upper limit of normal, *VIP* cisplatin, etoposide, ifosfamide

^a^Unknown / burned out tumor: cohort A: *n* = 0, cohort B: *n* = 2 (0.8%)

^b^“n” indicates a normal value

^c^IGCCCG classification not possible: cohort A: 7/70 NSem patients did not have a known LDH-value and were classified as good prognosis due to favorable clinical parameters; cohort B: 3/245 (1.2%) patients not able to be classified into IGCCCG prognosis group due to missing information

^d^250 U/l was selected as upper limit of normal (ULN) for serum-LDH. A cut-off of 1.5 × ULN was selected for classification into IGCCCG intermediate risk group

^e^Other: Cohort A: *n* = 6 patients defined as treated in SAKK trials (SAKK 01/10 and SAKK 01/18) with 1 cycle of EP + RT or 1 cycle of carboplatin + RT, 2 patients with RT alone and 1 patient with not further specified radiochemotherapy. Cohort B had 4 patients with primary high-dose chemotherapy, 2 patients being treated with multiple cycles of POMB/ACE after 1 cycle of EP, multiple patients treated within clinical trials (SAKK) and some regimes not further specified

### Management of relapsed and de novo metastatic GCC

Administered first-line treatment regimens for metastatic disease were similarly distributed in both cohorts. Most patients received BEP (67.9% in cohort A, 71.0% in cohort B), followed by etoposide and cisplatin (EP) (8.6% in cohort A, 11.0% in cohort B). Other treatment regimens included etoposide, ifosfamide and cisplatin (VIP); paclitaxel, ifosfamid and cisplatin (TIP), as well as study protocols or upfront surgery in a minority of patients. Residual tumor resection was performed in 14.8% of patients in cohort A and 34.5% in cohort B, possibly due to more prevalent NSem histology in cohort B. Post-chemotherapy radiation was performed in 13.2% vs. 5.4% in the cohorts A and B, respectively, usually within study protocols (Swiss Group for Clinical Cancer Research (SAKK) 01/10 and SAKK 01/18 studies) (Table 1).

### Clinical outcomes by histology and IGCCCG prognostic group

**Fig. 3:**
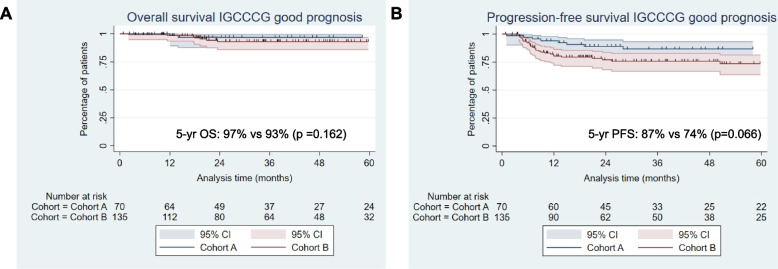
Kaplan–Meier curves illustrating OS and PFS for IGCCCG good prognosis relapsed (cohort A) from initial CSI vs de novo metastatic (cohort B) GCC patients. **A** 5-year OS was 97% vs 93% (*p* = 0.162) and **B** 5-year PFS, 88% vs 74% (*p* = 0.066) for cohort A vs B, respectively

High objective response rates were observed in both patient cohorts (98.7% in cohort A and 97.4% in cohort B). Complete response (CR) rates were numerically higher in cohort A (82.2% vs. 58.6%) (Table 1). Patients relapsed after initial CSI had better 5-year OS (94% vs 85%, *p* = 0.044) and PFS (86% vs 71%, *p* = 0.026), as compared to de novo metastatic patients (Suppl. Figure 2). When stratified by IGCCCG prognostic group, we observed a statistically non-significant trend towards better OS and PFS for good risk relapsed vs de novo metastatic patients (Fig. 3A, B). For this cohort of good risk patients, 5-year OS in cohort A vs B was 97% vs 93% (*p* = 0.162) and PFS 88% vs 74% (*p* = 0.066) (Fig. 3A, B). When stratified by histology, 5-year OS was 100% for relapsed Sem patients and 87% (95% CI: 61–96%) for relapsed NSems, respectively (Suppl. Figure 3A, B). During whole follow-up, no relapsed Sem and 4/40 (10%) NSem patients died (3 due to disease progression, 1 due to unrelated cause). 5-year PFS fsor relapsed CSI patients was 92% (95% CI: 77–97) for Sem and 78% (95%: 56–90) for NSem (Suppl. Figure 3C, D). 5-year OS and PFS rates for Sem and NSem patients stratified by IGCCCG prognostic groups are illustrated in the Suppl. Figures 3 and 4.

## Discussion

Within this work we report and analyze relapse data from a large cohort of consecutive CSI GCC patients from two university hospitals in Switzerland. For relapsed patients, we compared clinical features and survival outcomes to a second comparable cohort of de novo metastatic patients. In agreement with international guidelines, the majority of CSI GCC patients underwent AS following orchiectomy [4, 10]. In total, 22.5% of patients relapsed, which is in line with the 15–50% relapse rate reported by previous cohorts [11–18]. Previous studies correlated robustness and adherence to follow-up surveillance programs with excellent clinical outcome and low tumor burden in relapsed patients [19–21].

Our data show that CSI patients followed at our institutions most frequently relapsed within the good prognosis IGCCCG group and no patients relapsed with poor risk features. Relapsed CSI patients showed lower tumor marker levels and less non-pulmonary metastases compared to de novo metastatic patients. Eleven relapsed NSem patients were classified within the IGCCCG intermediate prognostic group uniquely due to increased serum LDH levels > 1.5 × ULN. Following the updated IGCCCG classification, 10 out of these 11 patients would have been classified within the good prognosis subgroup [5]. As expected, for relapsed Sem patients, we observed no deaths with a 100% OS at 5 follow-up vs 94% OS rate for de novo metastatic patients (*p* = 0.111). Similarly, for relapsed NSem patients, we observed a numerically better OS (87% vs 81%, *p* = 0.388) in favor of relapsed CSI patients.

In line with previous reports, the great majority of CSI patients relapsed within the first five years, which underlines the relevance of adherence to follow-up schedules within this time period [22, 23]. We did, however, observe 4.9% late relapses occurring after 5 years, all of which were initially managed with AS, which supports prolonged follow-up beyond the five- year critical follow-up period [24]. Data analysis from the Cancer Registry of Norway and Norwegian Cause of Death Registry showed that among patients with CSI GCC, late relapse (2–5 years) occurred in 1.9% of patients, very late relapse (5–10 years) in 1.0%, and extremely late relapse (> 10 years) in 0.5% [25]. The rates of late relapses were higher within the patient cohort managed by AS as compared to adjuvant treatment (4.0% vs 0.9%) [25]. These data support the recommendation to maintain a 10-year follow-up in patients undergoing AS.

The present analysis is limited by its retrospective, double-center, single country study design. However, the real world setting and the relatively large sample size supports the robustness of reported results. Our results underline excellent PFS and OS of CSI GCC patients undergoing AS following orchiectomy, as well as the relevance of adherence to international follow-up guidelines to ascertain early detection of relapses. However, relapse rates reported by previous studies, which can be as high as 50%, support adjuvant chemotherapy as an alternative option for patients with risk criteria or personal preferences. Moreover, as the relapse rates after adjuvant chemotherapy are extremely low, this can avoid greater short- and long-term toxicity derived from salvage chemotherapy with 3–4 cycles BEP in a subset of patients. Therefore, patients should be carefully informed of the benefits and disadvantages of both strategies.

## Conclusions

Our data confirm excellent survival probabilities for patients after CSI managed according to international guidelines. Relapses from CSI GCC were detected early and metastatic disease was diagnosed almost exclusively within the favorable IGCCCG prognostic group. Our data further support the choice of an active surveillance strategy for the majority of CSI GCC patients.

### Supplementary Information

**Additional file 1: Suppl. Figure 1.** Schematic diagram illustrating descriptive analysis for clinical stage I patients. **Suppl. Figure 2.** Kaplan-Meier curves illustrating OS and PFS for the entire cohort of relapsed from initial CSI (cohort A) vs de novo metastatic patients (cohort B). **Suppl. Figure 3.** Kaplan-Meier curves illustrating OS and PFS in GCC patients relapsed from initial CSI (cohort A) vs de novo metastatic patients (cohort B) stratified by histology. **Suppl. Figure 4.** Kaplan-Meier curves for OS in cohort A vs. cohort B stratified by histology and IGCCCG prognostic group. **Suppl. Figure 5.** Kaplan-Meier curves for PFS in cohort A vs. cohort B stratified by histology and IGCCCG prognostic group.

## Data Availability

The datasets used and/or analyzed during the current study are available from the corresponding author on reasonable request.

## Abbreviations

AFP: Alpha-fetoprotein
AS: Active surveillance
BEP: Bleomycin, etoposide and platinum
CR: Complete response
CRar: complete response after resection of residual tumor
CSI: Clinical stage I
EP: Etoposide and platinum
GCC: Germ cell cancers
HCG: Human chorionic gonadotropin
IGCCCG: International Germ Cell Consensus Classification
LDH: Lactate dehydrogenase
KM: Kaplan–Meier
NSem: Non-seminoma
OS: Overall survival
PD: Progressive disease
PFS: Progression free survival
PR: Partial response
SAKK: Swiss Group for Clinical Cancer Research
Sem: Seminoma
ULN: Upper limit of normal
