# Supplementary material for: Excellent survival in relapsed stage I testicular cancer
Source: BMC Cancer. 2023 Sep 15;23:870. doi: 10.1186/s12885-023-11388-y (PMC10503206; doi:10.1186/s12885-023-11388-y)
Supplement: Supplementary file 1 — Additional file 1: Suppl. Figure 1. Schematic diagram illustrating descriptive analysis for clinical stage I patients. Suppl. Figure 2. Kaplan-Meier curves illustrating OS and PFS for the entire cohort of relapsed from initial CSI (cohort A) vs de novo metastatic patients (cohort B). Suppl. Figure 3. Kaplan-Meier curves illustrating OS and PFS in GCC patients relapsed from initial CSI (cohort A) vs de novo metastatic patients (cohort B) stratified by histology. Suppl. Figure 4. Kaplan-Meier curves for OS in cohort A vs. cohort B stratified by histology and IGCCCG prognostic group. Suppl. Figure 5. Kaplan-Meier curves for PFS in cohort A vs. cohort B stratified by histology and IGCCCG prognostic group. [file 12885_2023_11388_MOESM1_ESM.pptx]

## Slide 1
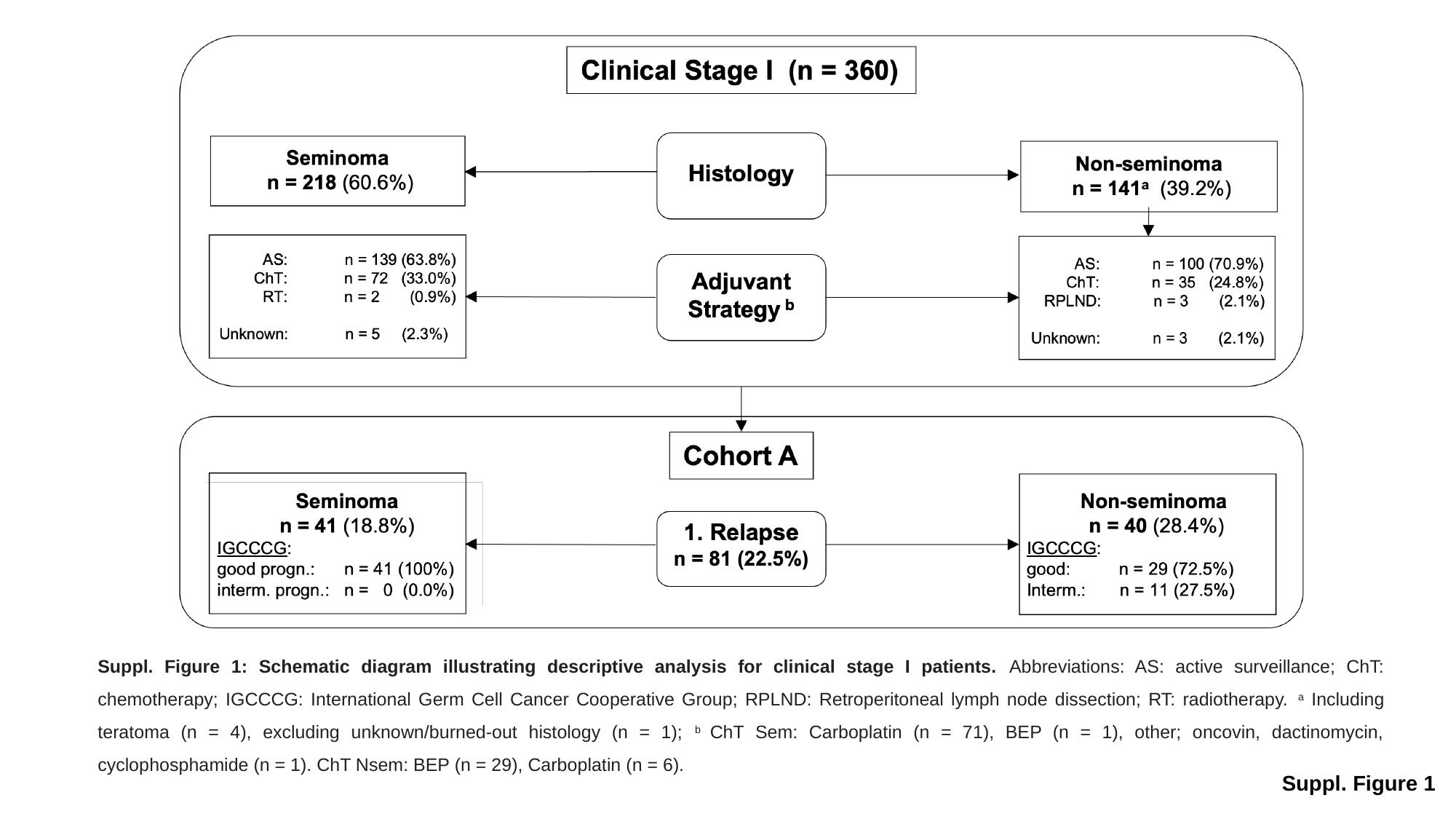

Suppl. Figure 1: Schematic diagram illustrating descriptive analysis for clinical stage I patients. Abbreviations: AS: active surveillance; ChT: chemotherapy; IGCCCG: International Germ Cell Cancer Cooperative Group; RPLND: Retroperitoneal lymph node dissection; RT: radiotherapy. a Including teratoma (n = 4), excluding unknown/burned-out histology (n = 1); b ChT Sem: Carboplatin (n = 71), BEP (n = 1), other; oncovin, dactinomycin, cyclophosphamide (n = 1). ChT Nsem: BEP (n = 29), Carboplatin (n = 6).
Suppl. Figure 1

## Slide 2
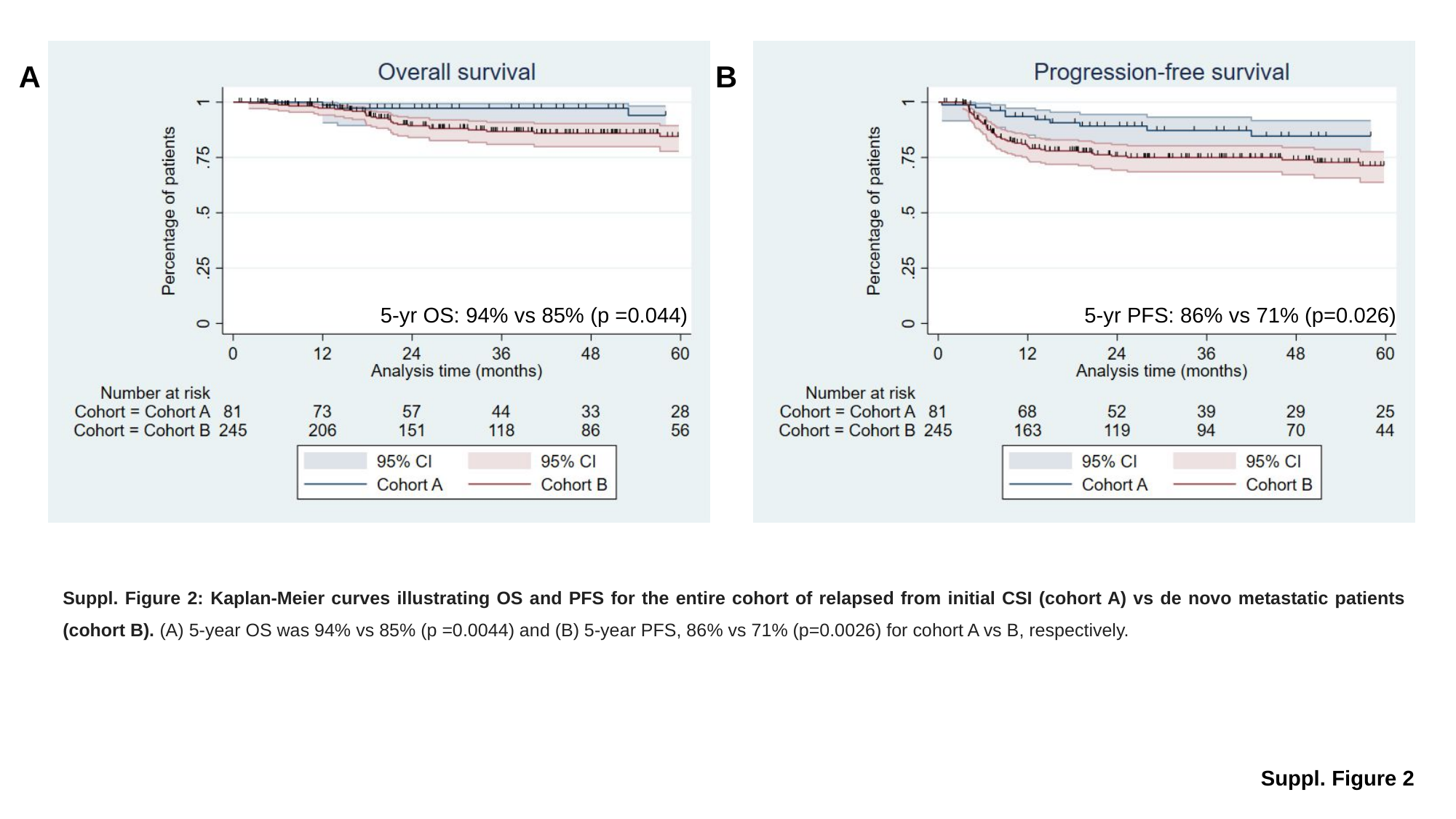

A
B
5-yr OS: 94% vs 85% (p =0.044)
5-yr PFS: 86% vs 71% (p=0.026)
Suppl. Figure 2: Kaplan-Meier curves illustrating OS and PFS for the entire cohort of relapsed from initial CSI (cohort A) vs de novo metastatic patients (cohort B). (A) 5-year OS was 94% vs 85% (p =0.0044) and (B) 5-year PFS, 86% vs 71% (p=0.0026) for cohort A vs B, respectively.
Suppl. Figure 2

## Slide 3
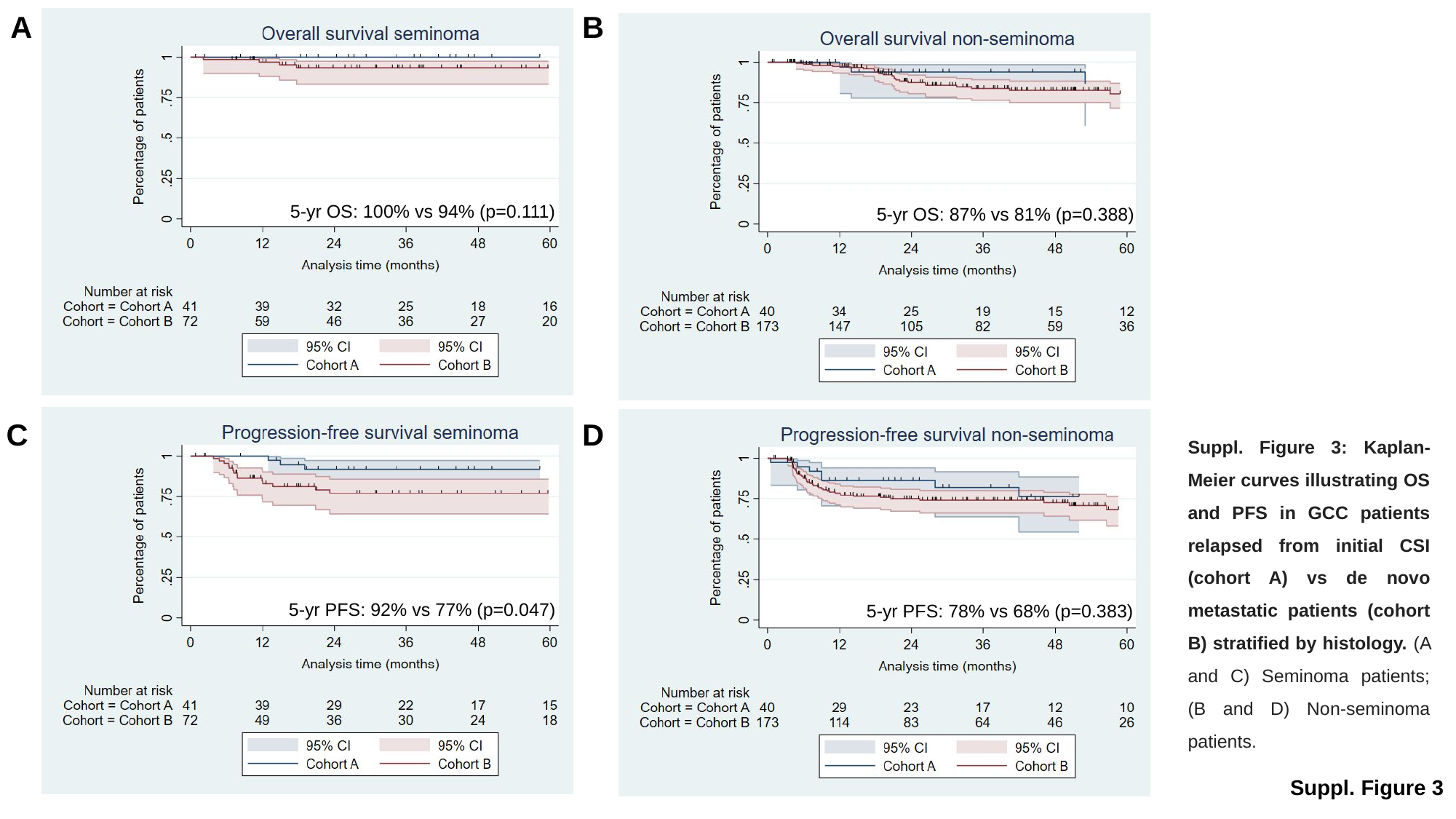

A
B
5-yr OS: 100% vs 94% (p=0.111)
5-yr OS: 87% vs 81% (p=0.388)
C
D
Suppl. Figure 3: Kaplan-Meier curves illustrating OS and PFS in GCC patients relapsed from initial CSI (cohort A) vs de novo metastatic patients (cohort B) stratified by histology. (A and C) Seminoma patients; (B and D) Non-seminoma patients.
5-yr PFS: 92% vs 77% (p=0.047)
5-yr PFS: 78% vs 68% (p=0.383)
Suppl. Figure 3

## Slide 4
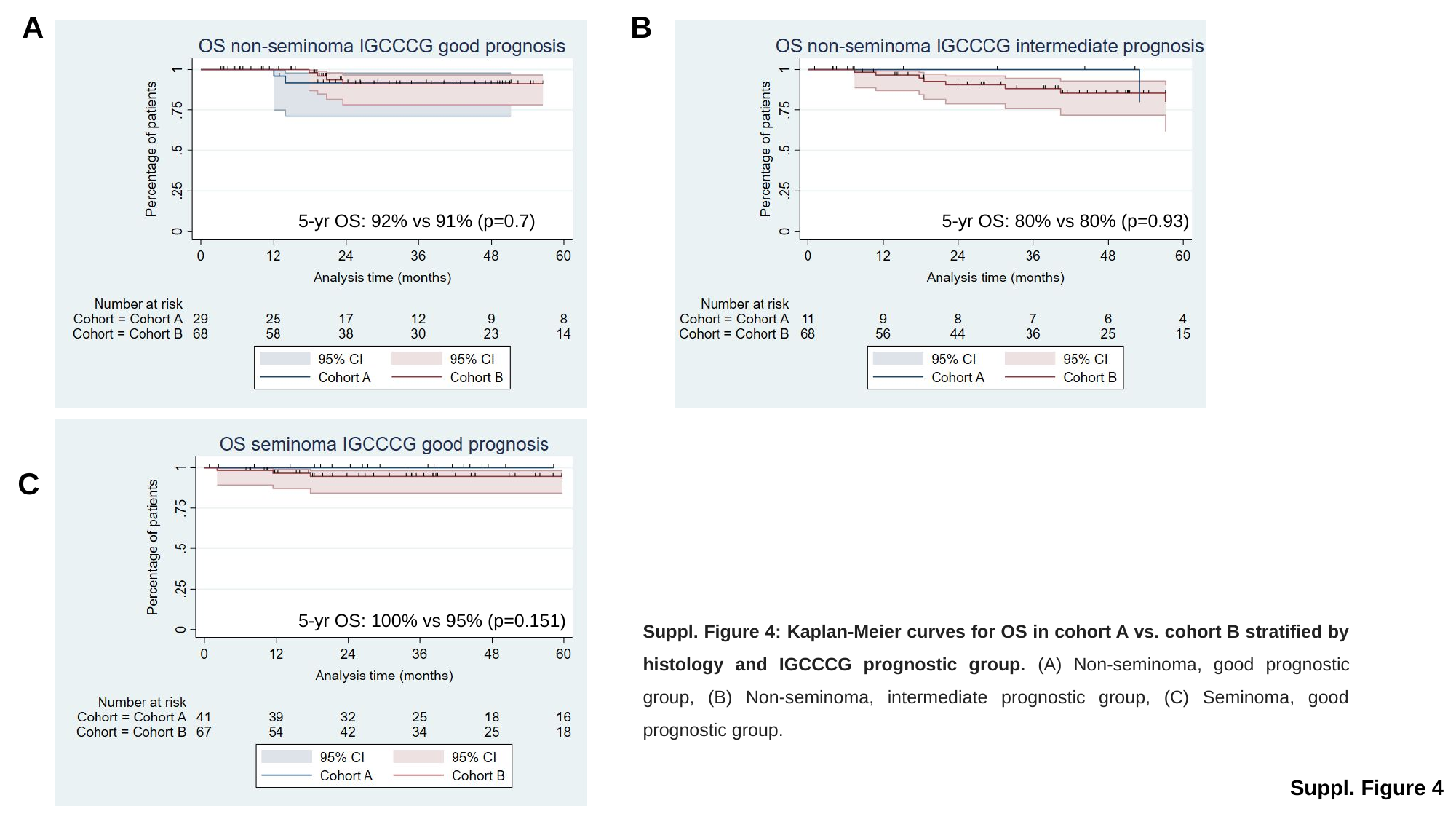

A
B
5-yr OS: 92% vs 91% (p=0.7)
5-yr OS: 80% vs 80% (p=0.93)
C
5-yr OS: 100% vs 95% (p=0.151)
Suppl. Figure 4: Kaplan-Meier curves for OS in cohort A vs. cohort B stratified by histology and IGCCCG prognostic group. (A) Non-seminoma, good prognostic group, (B) Non-seminoma, intermediate prognostic group, (C) Seminoma, good prognostic group.
Suppl. Figure 4

## Slide 5
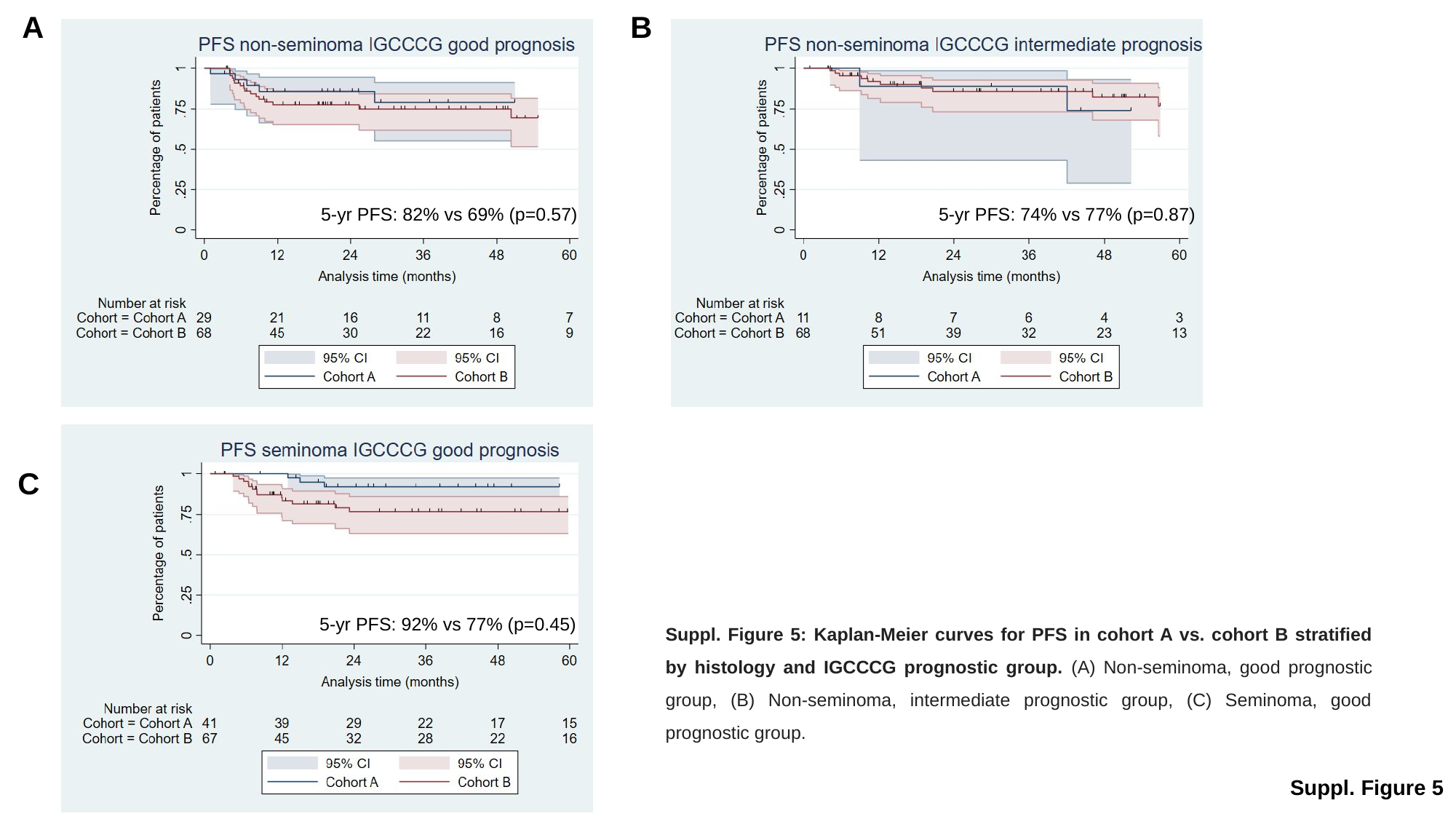

A
B
5-yr PFS: 82% vs 69% (p=0.57)
5-yr PFS: 74% vs 77% (p=0.87)
C
5-yr PFS: 92% vs 77% (p=0.45)
Suppl. Figure 5: Kaplan-Meier curves for PFS in cohort A vs. cohort B stratified by histology and IGCCCG prognostic group. (A) Non-seminoma, good prognostic group, (B) Non-seminoma, intermediate prognostic group, (C) Seminoma, good prognostic group.
Suppl. Figure 5
